# Menthacarin, a proprietary combination of peppermint and caraway oil, alters cultured human fecal microbiota composition, resulting in increased SCFA production

**DOI:** 10.3389/fphar.2025.1569052

**Published:** 2025-04-30

**Authors:** Martin D. Lehner, Philippe Ulsemer, Sandra Christochowitz

**Affiliations:** ^1^ Preclinical R&D, Dr. Willmar Schwabe GmbH & Co. KG, Karlsruhe, Germany; ^2^ ACARYON GmbH, Berlin, Germany

**Keywords:** microbiota, Menthacarin, peppermint, caraway, short-chain fatty acids, functional dyspepsia, irritable bowel syndrome, disorders of gut-brain interaction

## Abstract

**Background:**

Disruptions in the gut microbiota metabolism may contribute to the pathophysiology of gut–brain interaction disorders, and correction of intestinal dysbiosis is considered a promising therapeutic approach. Menthacarin, a proprietary fixed combination of *Mentha x piperita L*. and *Carum carvi L.* essential oils, is used clinically for the treatment of functional dyspepsia and irritable bowel syndrome. Rodent model data indicate that treatment effects of Menthacarin on visceral hypersensitivity could be mediated *via* the normalization of gut dysbiosis. However, the impact of Menthacarin on human bacterial gut microbiota has not yet been studied.

**Aim:**

The aim of the present study was to assess whether Menthacarin affects the composition and metabolic activity of human fecal microbiota.

**Methods:**

Fecal slurry samples from 10 healthy volunteers were cultivated for 36 h under anoxic conditions with and without Menthacarin. Relative bacterial abundance at the phylum and genus levels was evaluated using 16S rRNA metagenomic analysis. Short-chain fatty acids (SCFAs) in the supernatants were measured using the LC-MS technology.

**Results:**

Menthacarin induced robust changes in microbial composition at both the phylum and genus levels among the 10 donor microbiomes. The relative abundance of *Firmicutes* (+13.6 ± 8.6%) and *Actinobacteria* (+54.9 ± 47.6%) significantly increased, whereas that of *Bacteroidetes* (−27.7% ± 21.9%) and *Proteobacteria* (−25.7% ± 12.3%) significantly decreased in the presence of Menthacarin. At the genus level, the most notable changes were significant increases in *Bifidobacterium* (+105.1 ± 78.4%) and several SCFA-producing genera accompanied by a significant decrease in genera containing members involved in pro-inflammatory processes. In addition, Menthacarin significantly increased the levels of several SCFAs, namely, propionate, butyrate, isobutyrate, valerate, and isovalerate.

**Conclusion:**

Menthacarin alters the microbiota composition and enhances SCFA production in human microbiota samples under *in vitro* conditions. These effects may contribute to the clinical benefits observed with Menthacarin treatment.

## Introduction

Disorders of the gut–brain interaction (DGBIs), such as irritable bowel syndrome (IBS) and functional dyspepsia (FD), are debilitating gastrointestinal disorders. Although the etiology remains unknown, DGBIs are characterized by a combination of gastrointestinal motility disturbance, visceral hypersensitivity, altered mucosal and immune functions, or altered central nervous system processing. In addition, changes in the gut microbiota or dysbiosis have been implicated in the pathogenesis of DGBIs. Studies have shown that patients with IBS and FD often exhibit an altered gut microbiota composition compared to healthy individuals (Brown et al., 2022; Shanahan et al., 2023; Zhang et al., 2024b; Hillestad et al., 2022; Quigley, 2018; Gawey et al., 2024). For instance, a decrease in beneficial bacteria, such as *Lactobacillus* and *Bifidobacterium*, and an increase in potentially harmful bacteria have been observed in patients with IBS (Zhang et al., 2024c). However, different studies have produced inconsistent results, and to date, no defined disease-specific dysbiosis profile can be ascribed to patients with IBS (Teige et al., 2024) or FD (Zhou et al., 2022). Despite these limitations in defining a healthy versus disease-associated microbiota profile, treatments targeting microbiota composition and function are currently being studied or have already shown some efficacy in patients with DGBIs. These options include the use of prebiotics, probiotics, antibiotics, fecal transplantation, or fermentable oligo-, di-, and monosaccharides, and polyols diet (FODMAP) (Hillestad et al., 2022). In addition to these approaches specifically designed to address microbiota composition or function, the established pharmacological therapies for treating IBS and FD symptoms could also act by impacting the gut microbiota. This is especially relevant for phytotherapies based on mixtures of natural products, such as essential oils (EOs) from peppermint and caraway that are known to have antibacterial activity. Menthacarin is a proprietary combination of peppermint oil (90 mg WS^®^ 1340) and caraway oil (50 mg WS^®^ 1520) with specified quality. In clinical trials, Menthacarin has shown efficacy in reducing symptoms in patients with functional dyspepsia, including symptoms of IBS (Madisch et al., 2023; Madisch et al., 1999; Madisch et al., 2019; May et al., 2000; Rich et al., 2017; Holtmann et al., 2003). Human pharmacology studies have indicated that Menthacarin reduced gastroduodenal motility (Micklefield et al., 2000). Nonclinical mechanistic studies have confirmed the anti-spasmodic effects of both EOs on small and large human intestinal preparations in organ bath studies, mediated via calcium channel inhibition (Krueger et al., 2020). In addition, both EOs stimulate ion secretion in human intestinal preparations (Krueger et al., 2020). Studies in rodent models showed attenuation of intestinal inflammation by Menthacarin in a colitis model (Alliger et al., 2020) and normalization of visceral hypersensitivity induced by different noxious stimuli (Adam et al., 2006; Botschuijver et al., 2018; Omoloye et al., 2024). In maternally separated rats subjected to water avoidance stress, the induction of visceral hypersensitivity was associated with changes in the composition of the gut microbiome and mycobiome. Interestingly, the reversal of visceral hypersensitivity by Menthacarin was associated with normalization of the mycobiome, suggesting that effects on gut microbial or fungal composition could contribute to treatment effects of Menthacarin. Indeed, antimicrobial activities have been described for EOs, including peppermint oil (Hawrelak et al., 2009; Sharafi et al., 2010; Botschuijver et al., 2018; Neagu et al., 2023; Quintana Soares Lopes et al., 2024), caraway oil (Botschuijver et al., 2018; Hawrelak et al., 2009), and the combination product Menthacarin (Botschuijver et al., 2018), which exerted antibacterial and antimycotic activities in different *in vitro* assays. These data suggest that modulation of gut microbiota could contribute to the beneficial effects observed with Menthacarin or its individual constituents in nonclinical models and in patients with DGBIs. However, classical plate diffusion assays with isolated pathogens are of limited relevance to predict the effects of Menthacarin on human microbiome composition with a high level of mutual interactions and specific cultivation requirements. In addition, the effects observed in rodent microbiome samples may not be predictive of the human microbiome composition. To overcome these limitations, we employed an optimized anoxic culture system to assess the effect of Menthacarin on human microbiome composition and metabolic function in fecal samples from 10 healthy donors. Sequencing of 16S rRNA and mass spectrometric determination of SCFAs revealed consistent treatment effects of Menthacarin on microbiome composition, resulting in enhancement of SFCA production.

## Materials and methods

### Test items

Menthacarin^®^ is a proprietary combination of peppermint oil (90 mg WS^®^ 1340) and caraway oil (50 mg WS^®^ 1520) with specified quality. Menthacarin and individual EOs were provided by Dr. Willmar Schwabe GmbH & Co. KG. The EOs fulfilled the specification requirements of the European Pharmacopeia.

### *Ex vivo* cultivation

The present study was conducted using the HUMIPLATE-Platform (ACARYON GmbH, Berlin, Germany), a high-throughput *ex vivo* gut microbiome simulation model. It was designed to maintain the compositional stability of the microbiome for 48 h while also maintaining the functional capacities of the microbiome intact. Hence, the platform enables close replication of the gut microbiome’s *in vivo* dynamics in response to the test products.

In this study, feces from 10 healthy donors (five males and five females, Table 1) were used to assess the potential microbiome modulation properties of Menthacarin and its individual constituents, namely, peppermint oil (WS^®^ 1340) and caraway oil (WS^®^ 1520). The microbiomes were provided by ACARYON. The donors provided their samples at the doctor’s offices. Each donor signed a declaration of consent and a transfer of ownership. Strict data protection measures were observed to ensure that the samples could not be traced.

**TABLE 1:** Characteristics of fecal sample donors.

Microbiome	Gender	Age (years)	BMI
1	Male	54	22.7
2	Male	52	23
3	Male	45	33
4	Female	26	20.1
5	Female	54	30.4
6	Male	23	24.9
7	Female	27	17.8
8	Female	29	31.7
9	Female	50	31.6
10	Male	51	26

**FIGURE 1:**
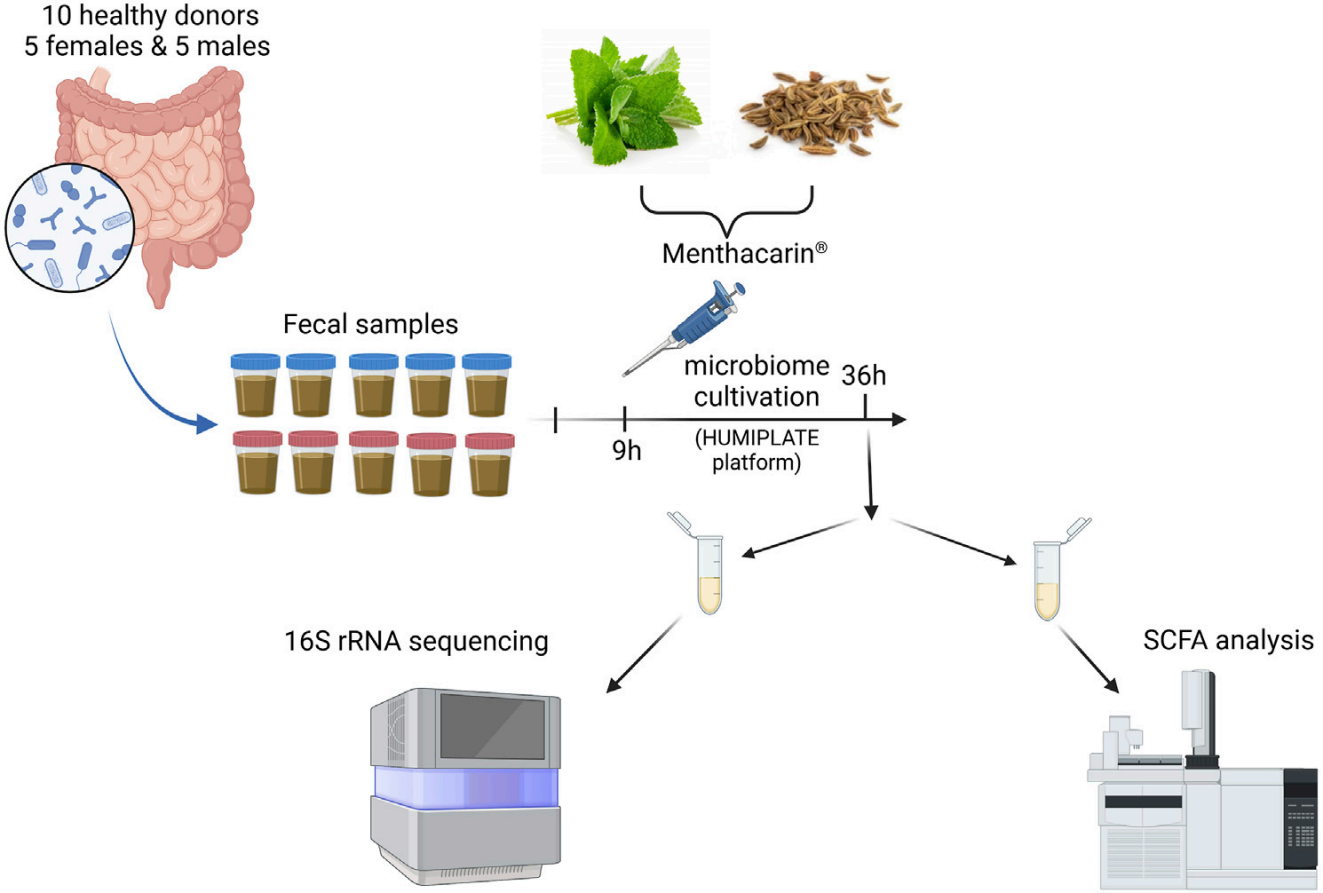
Experimental outline. Created in BioRender. Lehner M. (2025) https://BioRender.com/o98a156.

ACA-Medium (ACA-M001; ACARYON GmbH, Berlin, Germany) was inoculated at T0 with donor microbiomes. After 9 h (time for the microbiome to acclimate to the *ex vivo* conditions), Menthacarin or individual oils were added to each microbiome at a final concentration of 1 mg/mL. Each condition was run in duplicate. All experiments were performed in an anaerobic chamber. After 36 h, microbiomes were harvested. The microbiomes were first assessed for viability before centrifugation to separate the pellets from the cell-free supernatants. The pellets were subjected to 16S rRNA sequencing. The cell-free supernatants were stored at −80°C before being used for quantification of SCFAs (only vehicle and Menthacarin treatments) (Figure 1).

### Microbiome viability

The growth and viability of the microbiomes were controlled by determining the live/dead ratio within the culture using a LIVE/DEAD BacLight Bacterial Viability Kit (Thermo Fisher L7012). The assay was performed according to the manufacturer’s instructions.

### Next-generation sequencing

Next-generation sequencing (16S rRNA metagenomic analysis) was performed in a blinded manner.

### Sample storage and lysis

The collected samples were stored in a DNA-stabilizing buffer at −20°C until use. For the lysis process, the samples were defrosted and centrifuged at 4,000 g for 15 min. Later, 650 µL of warmed up PW buffer from the QIAamp 96 PowerFecal QIAcube HT Kit (Qiagen, Germany) were added to each sample and vortexed for 20 min.

### DNA extraction of stool samples

DNA was extracted with a proprietary in-house process using the QIAamp 96 PowerFecal QIAcube HT Kit on a liquid-handling system (Hamilton StarLine and Tecan EVO) using a vacuum chamber and a high-pressure chamber. The extracted gDNA was stored at −20°C until use.

### Library preparation for sequencing with the Illumina MiSeq System

The library preparation was performed following the manual “16S Metagenomic Sequencing Library Preparation—Preparing 16S Ribosomal RNA Gene Amplicons for the Illumina MiSeq System,” using the Master Mix (New England BioLabs) for the target and library amplification and using the 16S V3V4 primer (Eurofins). For the normalization of all samples, a fluorescent dye and the Biotek Synergy HTX plate reader were used to measure DNA concentrations and to calculate the necessary dilution volume per sample. All steps described were nearly fully automated using a liquid-handling system (Hamilton StarLine) that allows the processing of multiple samples simultaneously. Library Denaturing and MiSeq Sample Loading were carried out manually following the Illumina protocol for the MiSeq Reagent Kit v3 (600-cycle). Demultiplexing was performed directly on MiSeq after sequencing, and the resulting FastQ files were generated for subsequent data analysis.

### Processing and analysis of sequence data

Paired-end reads from MiSeq (2 × 300 cycles) were merged to reconstruct overlapping sequences with 430–460 base lengths. Chimera and borderline reads were filtered out with the usearch uchime2_ref tool (uchime2_ref command, drive5.com). SILVA 138.1 was used as the database for usearch uchime2_ref.

For taxonomic alignment, Amplicon sequence variants (ASVs) were determined using BLASTn (Nucleotide–Nucleotide BLAST 2.10.1+) against SILVA 138.1 (Release 138.1, arb-silva. de). Alignment identity was required to meet a threshold of at least the following: phylum, 75.0; class, 78.5; order, 82.4; family, 86.5; genus, 94.5; and species, 97.0. RefSeqs/Counts tables were created for all samples using Python package Pandas 1.3.4 (pandas-dev/pandas: Pandas 1.4.3 | Zenodo).

### SCFA quantification

Standards and supernatants of microbiome samples were derivatized with aniline, according to the method described by Haidacher et al. (2020). Calibration standards and samples were injected into a CSH Fluoro-Phenyl Column (130 Å, 1.7 µm, 2.1 mm × 150 mm). Acetonitrile (0.1% FA) and ULC-MS water (0.1% FA) were used as the mobile phase. Standards and samples were analyzed using a multiple reaction monitoring (MRM) MS method. This targeted method selects a specific MS2 fragment for quantification, which is generated by defined collision energies for each component. Acquired data of standards and samples were analyzed using Data Analysis (DA) software. Quantification was performed using the generated extracted ion chromatograms (EIC) based on MRM MS2 quantifier signals. The peaks were integrated, and the peak area was used to calculate the amount of SCFAs in each sample on the basis of the calibration curve.

### Bioinformatic processing

#### Copy number normalization

Further analyses were done using Picrust2 (Douglas et al., 2020). The study sequences of the alignment step were placed into a reference tree to determine/predict the copy numbers and the nearest-sequenced taxon index (NSTI index). All study sequences with an NSTI-index > 2 were excluded.

#### Assessment of alpha diversity

For alpha diversity calculations, ASV counts were rarefied to 10,000 counts per sample. The Shannon index was chosen as the alpha diversity metric, and calculations were performed using the “estimate_richness” diversity function provided by Phyloseq (McMurdie and Holmes, 2013). The R package ggplot (Villanueva and Chen, 2019) was used for visualization.

#### Assessment of beta diversity

To account for the compositional properties of relative ASV abundance, a total sum-scaling (TSS) transformation was performed for further analysis. This transformation was previously shown to outperform other transformative processes in distance-based analysis.

Bray–Curtis distances between the samples were obtained using “ordination” function in Phyloseq (McMurdie and Holmes, 2013). Compositional differences between the treatment groups were visualized *via* a principal component analysis, which was conducted using the PCA function of the R package Phyloseq (McMurdie and Holmes, 2013).

#### Assessment of treatment effects at phylum and genus levels

Variations in genera and phyla were illustrated *via* the log2 fold change of significantly different taxa. The R package ggplot2 (Villanueva and Chen, 2019) was used for visualization. For quantification of treatment effects, relative changes in abundance levels after test item treatment were expressed as % change vs. control treatment. Heatmaps and graphs containing % change data were generated in GraphPad Prism version 10.1.2 for Windows (GraphPad Software, Boston, Massachusetts, United States, www.graphpad.com).

### Statistical analysis

#### Alpha diversity

An unpaired two-sided *t*-test was performed for hypothesis testing, and the calculated p-values were adjusted for multiple testing, using the Benjamini and Hochberg correction method (Benjamini and Hochberg, 1995).

#### Beta diversity

For statistical analysis of beta diversity, Permanova analysis was performed using the adonis2 function from the R package vegan (Oksanen and Blanchet, 2022) and pairedAdonis2 package; the resulting p-values were adjusted for multiple testing using the Benjamini and Hochberg correction method (Benjamini and Hochberg, 1995).

#### Phyla and genera abundance

To identify differentially abundant taxa between the samples with and without test items, a two-sided paired Wilcoxon signed-rank test was performed with the following:

i) centered-log ratio normalized relative abundances and
ii) relative abundance without test product normalization to 1: the relative abundances in the controls were set to 1, and the relative abundances obtained following cultivation with the product were proportionally normalized.

Statistical significance was set at a p-value <0.05. Analysis was carried out at the genus and phylum levels while only considering samples for statistical comparison with a mean abundance of more than 0.15% in at least one group (with or without Menthacarin).

#### SCFA quantification

For statistical analysis of treatment effects on SCFA levels, the Wilcoxon matched pairs signed-rank test for comparison of control vs Menthacarin treatment was performed using GraphPad Prism version 10.1.2 for Windows (GraphPad Software, Boston, Massachusetts United States, www.graphpad.com).

## Results

### Microbiome diversity

**FIGURE 2:**
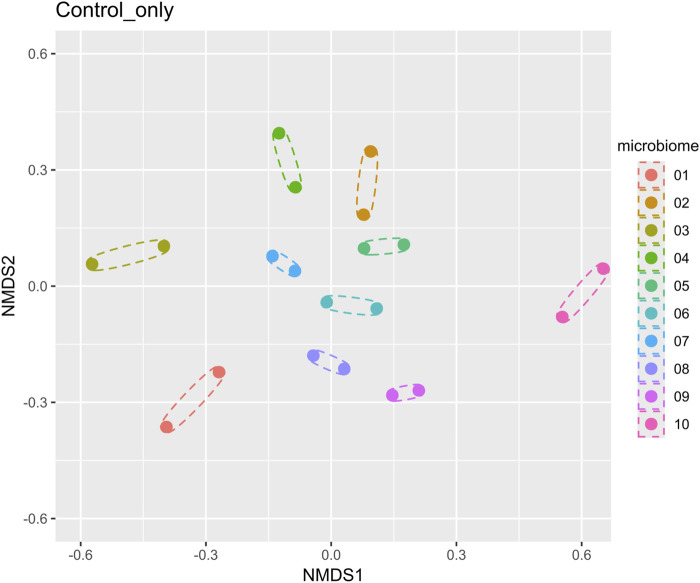
Beta diversity analysis of 10 microbiomes (without Menthacarin) using a nonmetric multidimensional scaling method. Each microbiome has two replicates, which are shown as dotted ellipses.

Ten microbiomes from 10 healthy donors, 5 males and 5 females of different ages and BMIs, were used in the study (Table 1). The analysis of beta diversity assessed using Bray-Curtis distances and visualized through the principal coordinate analysis plot (PCoA) demonstrated that the 10 microbiomes provided an appropriate diversity for the study (Figure 2).

### Effect on alpha diversity

**FIGURE 3:**
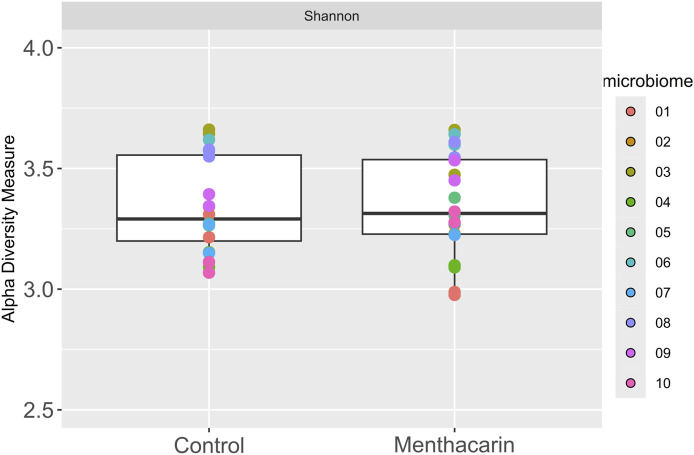
Alpha diversity analysis of 10 microbiomes with and without Menthacarin using Shannon’s index as a metric.

After 36 h of cultivation with Menthacarin, the viability of the microbiomes was above 90% in all individual microbiome samples (Supplementary Table S1), demonstrating that Menthacarin had no toxic effect on the human microbiome. Furthermore, no statistical difference between the microbiomes cultivated with and without Menthacarin could be observed in terms of alpha diversity, demonstrating that Menthacarin did not impact species richness (Figure 3).

As the study was run with 10 microbiomes, considering the very low abundance and/or low prevalence, taxa may have generated false-positive results. Consequently, to ensure the validity of the results, two successive filters were applied to select the taxa to be considered. First, for each taxon, only microbiomes presenting a relative abundance of at least 0.15% (at the considered taxa level) with or without Menthacarin were considered. Furthermore, only taxa for which at least four of the ten microbiomes fulfilled this condition were considered.

### Microbiome modulation at phylum level

**FIGURE 4:**
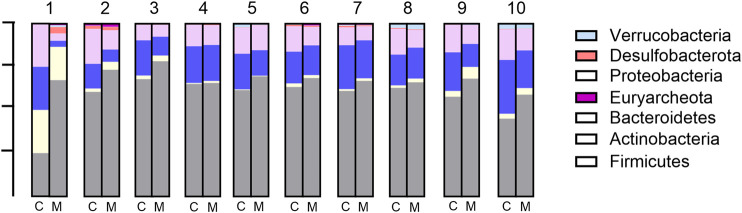
Microbiome composition at the phylum level: relative abundance (%) in control (C) vs Menthacarin (M)-treated microbiomes from 10 donors (1–10).

At the phylum level, the seven most abundant phyla were found to fit the selection criteria discussed above. In the control samples, *Firmicutes* was the most abundant phylum (mean: 60.4% and range: 45%–68%), followed by *Bacteroidetes* (mean: 20.1% and range: 9%–31%), *Proteobacteria* (mean: 13.8% and range: 7%–20%), and *Actinobacteria*, the latter being highly variable between different donor samples (mean: 3.1% and range: 0.4%–15.6%). *Desulfobacterota* (mean: 1.1% and range: 0.1%–5.9%), *Verrucobacteria* (mean: 1.0% and range: 0.001%–2.7%), and *Euryarchaeota* (mean: 0.4% and range: 0%–0.9%) were present only in low relative abundance in most samples (Figure 4). The addition of Menthacarin generally maintained the donors’ specific composition while increasing the relative abundance of *Firmicutes* and *Actinobacteria*, and decreasing the relative abundance of *Bacteroidetes* and *Proteobacteria* (Figure 4; Supplementary Figure S1).

**FIGURE 5:**
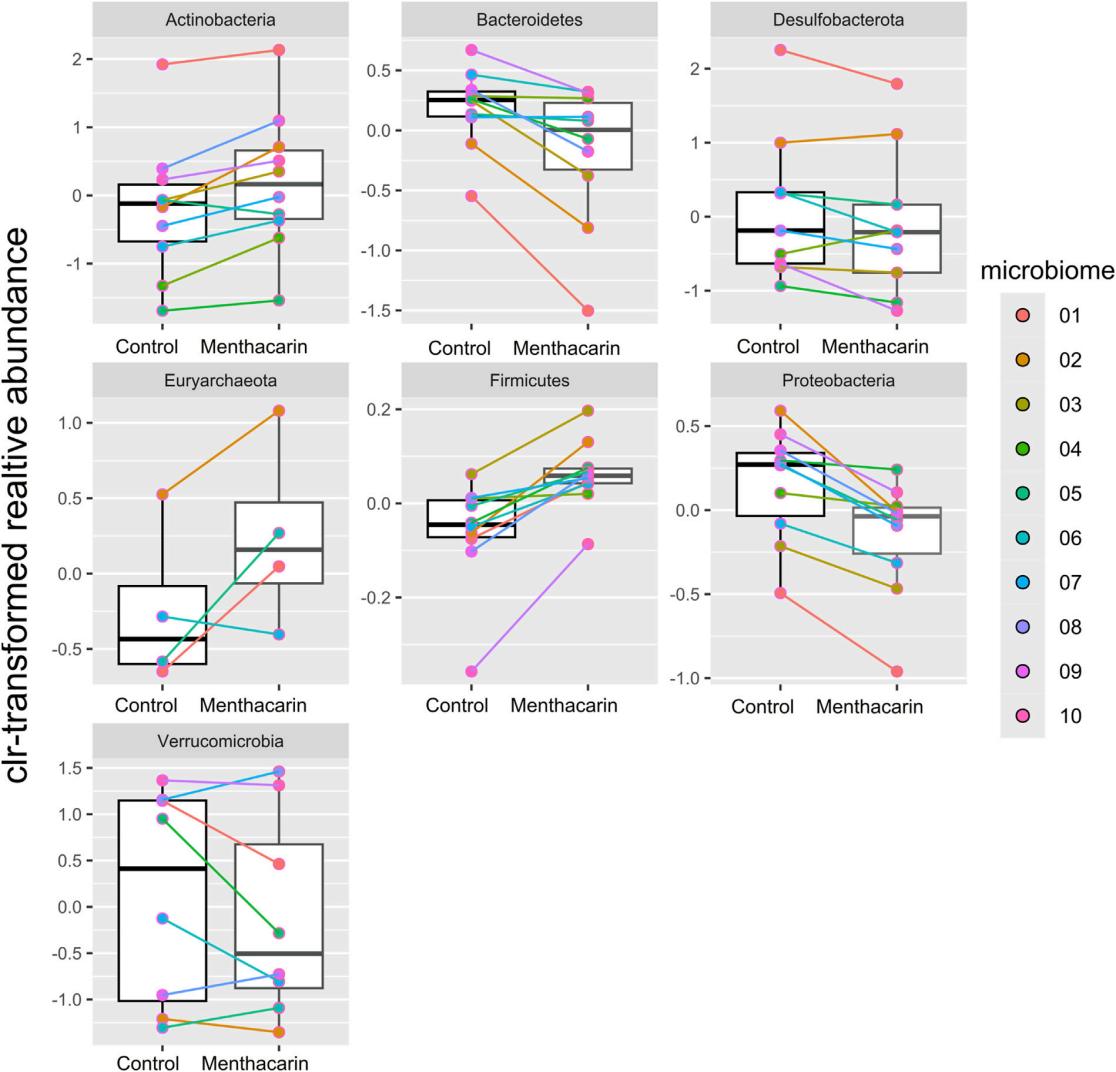
Comparison of centered log-ratio transformed relative abundance between the control and Menthacarin across the considered phyla. Box plots show median, quartile, and min–max with individual microbiome values highlighted in different colors. Individual lines connect corresponding data points for individual microbiomes with and without the addition of Menthacarin. Significant differences between control and Menthacarin treatments were found for *Actinobacteria* (p = 0.0059), *Bacteroidetes* (p = 0.0039), *Firmicutes* (p = 0.0020), and *Proteobacteria* (p = 0.0020) based on paired two-tailed Wilcoxon’s signed rank-sum test.

Menthacarin was found to induce a statistically significant increase in the relative abundance of the phyla *Firmicutes* (p = 0.0020) and *Actinobacteria* (p = 0.0059), and a statistically significant decrease in the relative abundance of the phyla *Bacteroidetes* (p = 0.0039) and *Proteobacteria* (p = 0.0020) when centered log-ratio transformed relative abundance was used for statistical analysis (Figure 5). Furthermore, although not statistically significant, Menthacarin decreased the relative abundance of the phylum *Desulfobacterota* in 7 out of 9 considered microbiomes (p = 0.0972; Figure 5).

**FIGURE 6:**
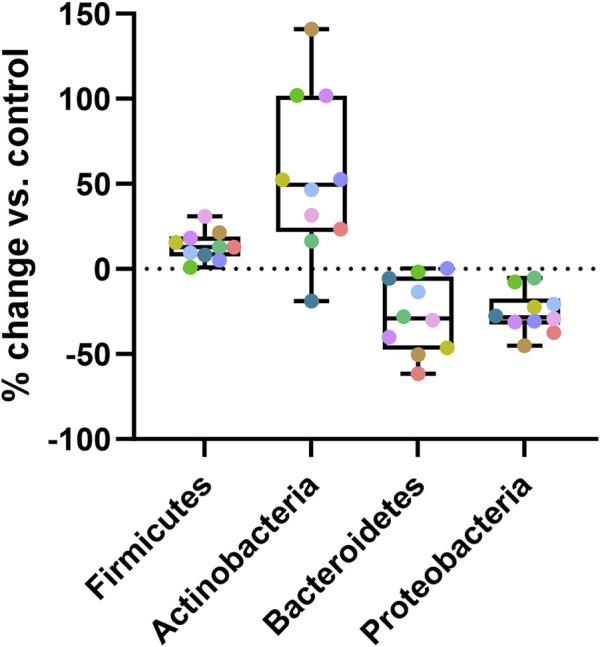
Menthacarin increases *Firmicutes* and *Actinobacteria* while decreasing *Bacteroidetes* and *Proteobacteria*. Percentage change in the relative abundance at the phylum level induced by Menthacarin (compared to control conditions) is shown. Box and Whiskers plot is presented with quartiles and min–max. Each colored point represents an individual microbiome.

For quantification of the effects of Menthacarin on the microbiome composition, we calculated the percentage change induced by Menthacarin in the relative abundance of the four phyla with significant treatment effects. The addition of Menthacarin led to an increase in *Firmicutes* and *Actinobacteria* by 13.6% ± 8.6% and 54.9% ± 47.6%, whereas it led to a decrease in *Bacteroidetes* and *Proteobacteria* by 27.7% ± 21.9% and 25.7% ± 12.3%, respectively (Figure 6).

### Microbiome modulation at genus level

**FIGURE 7:**
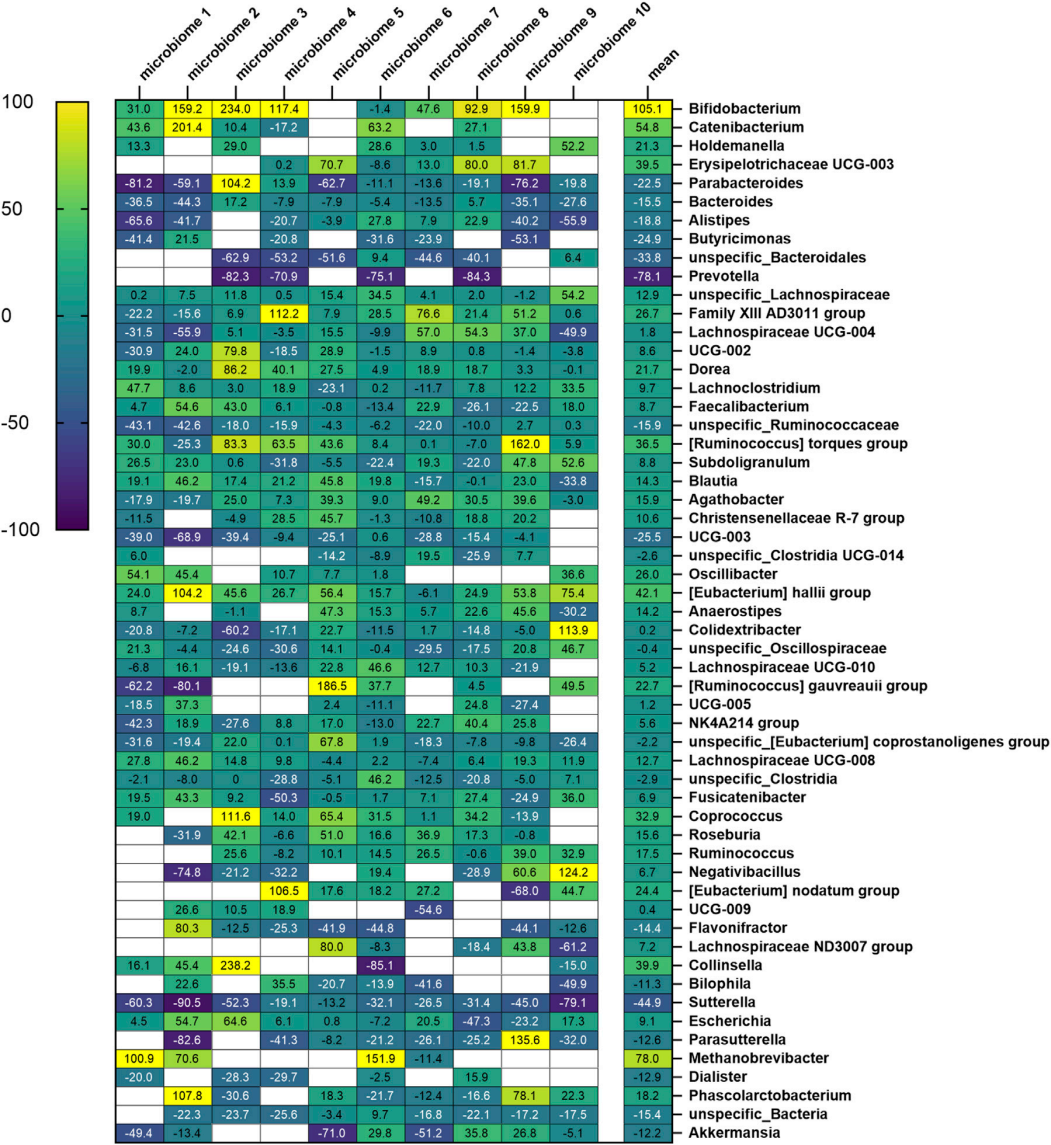
Heatmap of fractional change in relative abundance with Menthacarin (expressed in percentage) across all considered genera. The numbers in each cell denote the fractional change (in %) for each genus across the 10 microbiomes, with the mean values shown in the final column.

**FIGURE 8:**
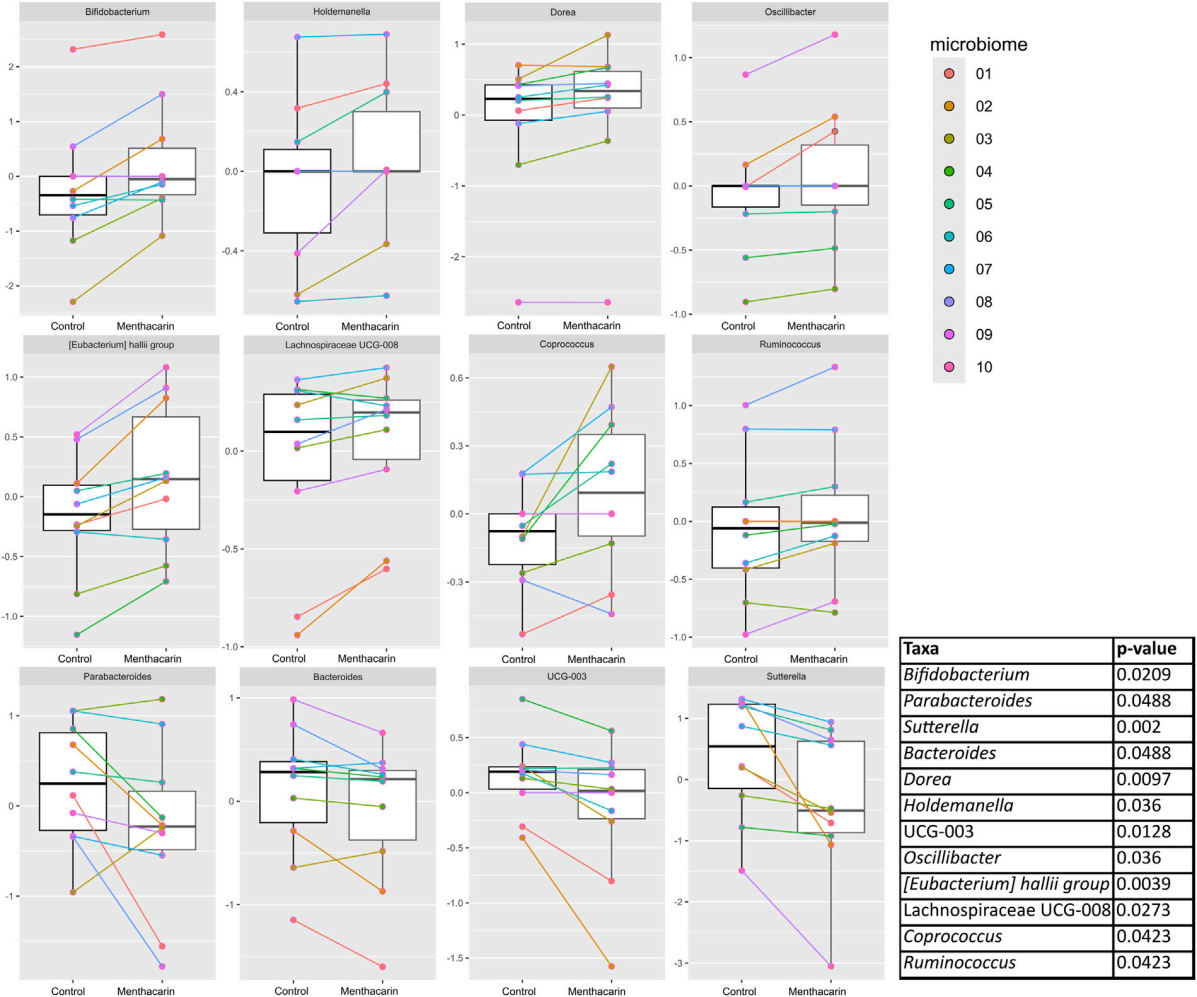
Comparison of centered log-ratio transformed relative abundance between the control and Menthacarin in statistically significantly different genera. Individual lines connect the corresponding data points for individual microbiomes with and without the addition of Menthacarin. A paired two-tailed Wilcoxon’s signed rank-sum test was used for statistical analysis with a p-value <0.05 considered statistically significant.

At the genus level, 173 specific genera (without considering “unclassified”) with relative abundance of at least 0.1% were identified across all 10 microbiomes (Supplementary Figure S2). After filtering, 52 specific genera were considered (Figure 7). For 35 of them, the effects of Menthacarin were qualitatively consistent among all or most of the 10 microbiomes (at least 70% of the considered microbiomes), and 12 presented a statistically significant change in response to Menthacarin (Figure 8).

### Menthacarin increases health-promoting and SCFA-producing genera

Menthacarin induced a strong (observed in 7 of 8 considered microbiomes, 105.1% ± 78.4% increase) and statistically significant increase in the relative abundance of the health-promoting genus *Bifidobacterium* (Figures 7, 8).

In accordance with the statistically significant increase observed in the phylum *Firmicutes*, Menthacarin was also found to promote several genera belonging to the classes *Clostridia* and *Bacilli*. Menthacarin induced a statistically significant increase in the relative abundance of the butyrate-producing genera *Eubacterium hallii*, *Coprococcus*, and *Ruminococcus* (Figures 7, 8). Furthermore, although not statistically significant, Menthacarin induced an increase in the relative abundance of the butyrate-producing genera *Blautia*, *Ruminococcus torque*, and *Anaerostipes* in 7 of 10, in 7 of 10, and in 6 of 8 considered microbiomes, respectively (Figure 7). Altogether, Menthacarin induced an increase in the relative abundance of the main butyrate producers of the class *Clostridia* (*Faecalibacterium*, *Eubacterium hallii*, *R. torque*, *Blautia*, *Anaerostipes*, *Coprococcus*, *Roseburia Ruminococcus*, and *Subdoligranulum*) (Figure 7). Menthacarin also significantly increased the relative abundance of the genera Lachnospiraceae UGC-008, *Oscillobacter*, and *Dorea* (Figure 7).

Moreover, Menthacarin promoted members of the class *Bacilli*. Menthacarin induced a statistically significant increase in the relative abundance of the genus *Holdemanella* and an increase in the relative abundance of the genera *Erysipelotrichaceae* UCG-003 and *Catenibacterium* in 5 of 6 considered microbiomes (Figures 7, 8).

### Menthacarin inhibits inflammation-related genera

At the genus level, five specific genera belonging to the phylum *Bacteroidetes* were considered. The relative abundance of the genera *Bacteroides* and *Parabacteroides* was significantly reduced by Menthacarin (Figure 8). Also, the relative abundance of the genera *Butyricimonas*, *Alistipes*, and *Prevotella* mostly decreased; however, the decrease was not statistically significant (Figure 7).

Among the phylum *Proteobacteria*, two members of the class *Gammaproteobacteria* were inhibited by Menthacarin. The relative abundance of the genus *Sutterella* was significantly reduced in the presence of Menthacarin, whereas for the genus *Parasutterella*, the relative abundance was decreased in 7 of 8 considered microbiomes (Figures 7, 8).

### Effects of peppermint and caraway EOs

Treatment of microbiomes with peppermint oil or caraway oil at 1 mg/mL produced qualitatively and quantitatively similar changes in the relative abundance at the phylum level with increases in *Firmicutes* and *Actinobacteria* and decreases in *Bacteroidetes* and *Proteobacteria* (Supplementary Figure S3). At the genus level, 17 genera were consistently affected (in at least 70% of tested microbiomes) by all three treatments: 9 were affected by Menthacarin and caraway oil, 2 by Menthacarin and peppermint oil, and 6 by peppermint and caraway oil. In addition, 7 genera were only consistently affected by Menthacarin, whereas 2 and 4 genera were uniquely affected by peppermint and caraway oil, respectively (Supplementary Figure S4).

### Menthacarin increases SCFA levels

**FIGURE 9:**
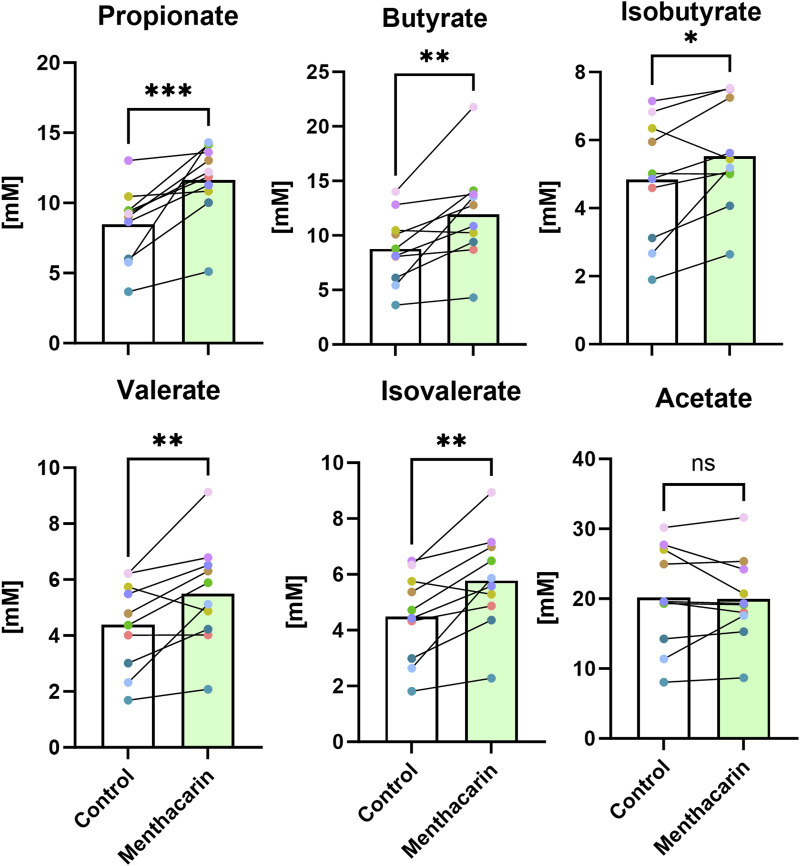
Menthacarin increases the concentration of most SCFAs. Concentrations of short-chained fatty acids in supernatants of control and Menthacarin-treated microbiomes are shown as medians of 10 microbiomes and individual microbiome values. Individual lines connect corresponding data points for individual microbiomes without (control) vs with the addition of Menthacarin. Statistical significance assessed by Wilcoxon matched pairs signed rank test for comparison of control vs. Menthacarin treatment is indicated by asterisks (*p < 0.05, **p < 0.01, and ***p < 0.001).

We observed a positive effect of Menthacarin on the relative abundance of several SCFA-producing genera. To assess whether the change in microbial composition translated into altered microbiome metabolite production, we analyzed the concentrations of different SCFAs in the supernatants of the microbiome cultures. As expected from the microbiome changes, Menthacarin significantly increased the production of propionate, butyrate, isobutyrate, valerate, and isovalerate, whereas no effect was observed on acetate levels (Figure 9).

## Discussion

Menthacarin, a proprietary combination of EOs from *Mentha x piperita L*. and *Carum carvi L.*, has demonstrated clinical efficacy in alleviating symptoms and improving the quality of life in patients with FD (Madisch et al., 2023; Madisch et al., 1999; Rich et al., 2017; Holtmann et al., 2003; Storr and Stracke, 2023) and concomitant IBS symptoms (Madisch et al., 2019). In a rat model of maternal separation and water avoidance stress, Menthacarin normalized the visceral hypersensitivity. This visceral hypersensitivity was associated with changes in the gut microbiome and mycobiome, which were partially corrected by Menthacarin, suggesting that modulation of gut microflora may be a potential mechanism of action (Botschuijver et al., 2018). In the present *in vitro* study, we assessed the effect of Menthacarin on human microbiome composition and metabolic activity. Using an optimized anoxic incubation protocol of fecal microbiome samples from 10 healthy donors, we observed that Menthacarin induced consistent changes in the microbiome composition among donors. Whereas alpha diversity remained unaffected, Menthacarin significantly increased the relative abundance of the phyla *Firmicutes* and *Actinobacteria*, and decreased that of *Bacteroidetes* and *Proteobacteria* (Figures 4–6). At the genus level, Menthacarin significantly increased the abundance of health-promoting bacteria such as *Bifidobacterium* and SCFA-producing genera within the class *Clostridia* (e.g., *Coprococcus*, *Ruminococcus torques*, *Dorea*, and *Eubacterium Hallii*) and the class *Bacilli* (*Holdemanella*) (Facchin et al., 2024). Conversely, genera from the phyla *Bacteroidetes* and *Proteobacteria*, which are considered to be inflammation-related, were significantly (*Bacteroides*, *Parabacteroides*, and *Sutterella*) or numerically decreased (*Butyricimonas*, *Alistipes*, *Prevotella*, and *Parasutterella*) (Figures 7, 8).

Although the 10 microbiomes displayed different microbiota compositions (Figures 2, 4), the effects of Menthacarin on the four main phyla and most genera mentioned above were qualitatively consistent among all or most microbiomes.

The robustness of the observed microbiota changes was confirmed by testing the single oil constituents of Menthacarin, namely, peppermint and caraway EOs, at the same concentration of 1 mg/mL. Both single EOs produced qualitatively and quantitatively comparable results as Menthacarin on microbiome composition, with increases in *Firmicutes* and *Actinobacteria*, as well as corresponding decreases in *Bacteroidetes* and *Proteobacteria* (Supplementary Figure S3). Furthermore, among the 35 genera with qualitatively consistent changes in response to Menthacarin, 17 also presented similar changes in response to peppermint oil and caraway oil (Supplementary Figure S4).

One of the most significant recent paradigm shifts in medicine is the recognition of the central role of gut microbiome in human health. The gut microbiome influences almost every aspect of human health and significantly impacts the effectiveness of drugs and treatments (Salvucci, 2019; Hou et al., 2022; Wilkinson et al., 2018; Weersma et al., 2020). Consequently, modulating the composition or functionality of the microbiome has become the main avenue for the prevention and treatment of diseases (Dietert, 2021; Yadav and Chauhan, 2022).

The connection between the gut microbiome and disturbed well-being is particularly relevant for disorders of gut–brain interactions such as IBS and FD (Brown et al., 2022; Gawey et al., 2024; Hillestad et al., 2022; Quigley, 2018; Shanahan et al., 2023; Teige et al., 2024; Zhang et al., 2024b; Zhang et al., 2024c; Zhou et al., 2022; Margolis et al., 2021). Several changes in the microbiome composition have been reported in patients with IBS, including a lower abundance of *Firmicutes* (Zhuang et al., 2018) and *Bifidobacterium* (Liu et al., 2017; Zhang et al., 2024c; Kamp et al., 2024) and higher levels of *Bacteroides* (Zhang et al., 2024c; Kamp et al., 2024; Zhuang et al., 2018) than in healthy controls. In addition, clinical studies have reported beneficial effects of treatments based on live or inactivated *Bifidobacterium* strains in patients with IBS and FD (Cruchet Muñoz et al., 2024; Srivastava et al., 2024; Zhang et al., 2024a). *Ex vivo* systems using microbiome samples have demonstrated the ability to reproduce most of the changes identified *in vivo*. For example, the *in vivo* effect of a drug like metformin on the murine microbiome has been shown to correlate with effects observed in an *ex vivo* system (Li et al., 2019). Another example demonstrated that *ex vivo* systems could simulate the clinical outcomes of repeated prebiotic intake (Van den Abbeele et al., 2023). We employed an *ex vivo* high-throughput cultivation system (HUMIPLATE, ACARYON GmbH, Germany) that was optimized to maintain the composition and functionality of the microbiome for 48 h. In our experimental setting, Menthacarin produced an increase in *Bifidobacterium* and reduced the abundance of *Bacteroides*, suggesting that the microbiome changes induced by Menthacarin have the potential to correct dysbiosis in patients with DGBI.

One limitation of our study design was the use of fecal samples only from healthy donors. Further studies with Menthacarin treatment of microbiomes from patients with DGBI are required to assess the normalization of disease-related dysbiosis. In our study, we tested Menthacarin at a concentration of 1 mg/ml). A single dose of the finished herbal medicinal product contains 140 mg of Menthacarin. Assuming an intestinal volume of 250 mL, a value applied for assessing the intestinal drug–drug-interaction potential (EMA/CHMP/ICH/652460/2022, 2022), a single dose application would result in a luminal concentration of approximately 0.56 mg/mL, suggesting that our test concentration is within a physiologically relevant range.

Extrapolating from microbiome composition data to actual health benefits is challenging due to the complexity of the microbiota and microbiota–host interactions. One approach to reduce this complexity is to analyze the effects on metabolite production, which serves as a common final pathway of microbe–host interactions. Short-chain fatty acids have attracted significant attention as products of microbial metabolism with well-documented beneficial effects on the host cell function and health. Among the activities most relevant for DGBI, SCFAs serve as an energy source for colonocytes and enhance mucus production, which protects the gut lining. In addition, SCFAs, particularly butyrate, have been shown to reduce inflammation and enhance the integrity of the gut barrier (Xiong et al., 2022). In our study, we found significant increases in the levels of all measured SCFAs (propionate, butyrate, isobutyrate, valerate, and isovalerate) after Menthacarin treatment, except for acetate, where no difference was observed (Figure 9). The increase in SCFA production suggests that the observed increases in genera known to be relevant for SCFA production indeed translate into metabolic changes. For patients with DGBI, SCFAs could not only exert beneficial effects by reducing intestinal permeability and inflammation but could also contribute to certain symptoms, especially diarrhea and bloating, depending on the individual situation. Interestingly, the administration of encapsulated butyrate has been shown to reduce symptom severity in patients with IBS and inflammatory bowel diseases (Facchin et al., 2020; Banasiewicz et al., 2013). Additionally, fecal microbiota transplantation in patients with IBS resulted in improvements in symptom scores and quality of life while increasing fecal butyrate, isobutyrate, isovalerate, and valerate levels (El-Salhy et al., 2021). In that study, butyrate levels were inversely correlated with symptom severity in responders, indicating a beneficial effect of (selected) SCFA increase in patients with IBS (El-Salhy et al., 2021).

Our *in vitro* cultivation system in healthy human microbiomes demonstrates that Menthacarin has the potential to induce changes in the relative microbiome composition, resulting in increased SCFA production. It is tempting to speculate that microbiome modulation could contribute to symptom reductions observed with Menthacarin therapy in animal models of visceral hypersensitivity and patients with FD. However, further nonclinical *in vivo* studies and patient microbiome data would be needed to verify this hypothesis.

In conclusion, incubation of human fecal samples with Menthacarin produced consistent changes in microbiome composition, characterized by an increase in *Firmicutes* and *Actinobacteria* and a decrease in *Bacteroidetes* and *Proteobacteria*. In addition, levels of SCFAs were significantly increased by Menthacarin. The data provide initial evidence that microbiome modulation could be a mechanism of action with potential relevance for the observed clinical efficacy of Menthacarin in patients with DGBI.

## Data Availability

The original contributions presented in the study are publicly available. This data can be found here: NCBI BioProject number PRJNA1250407, available at: https://www.ncbi.nlm.nih.gov/sra/PRJNA1250407.
