# Supplementary material for: Menthacarin, a proprietary combination of peppermint and caraway oil, alters cultured human fecal microbiota composition, resulting in increased SCFA production
Source: Front Pharmacol. 2025 Apr 30;16:1569052. doi: 10.3389/fphar.2025.1569052 (PMC12075938; doi:10.3389/fphar.2025.1569052)
Supplement: Supplementary file 1 [file DataSheet1.PDF]

Supplementary Material

Menthacarin, a proprietary combination of peppermint and caraway oil alters cultured human fecal microbiota composition resulting in increased SCFA production

Martin D. Lehner[1]*, Philippe Ulsemer[2], Sandra Christochowitz[1]

[1]Preclinical R&D, Dr. Willmar Schwabe GmbH & Co. KG, Karlsruhe, Germany

[2]ACARYON GmbH, Berlin, Germany

Supplementary Table S1: Viability analysis of the control cultures after incubation period (T36)

**Microbiome**	1	2	3	4	5	6	7	8	9	10
**Viability [%]**	93.3	92.4	92.5	91.2	92.2	90.8	91.7	92.2	91.0	92.8

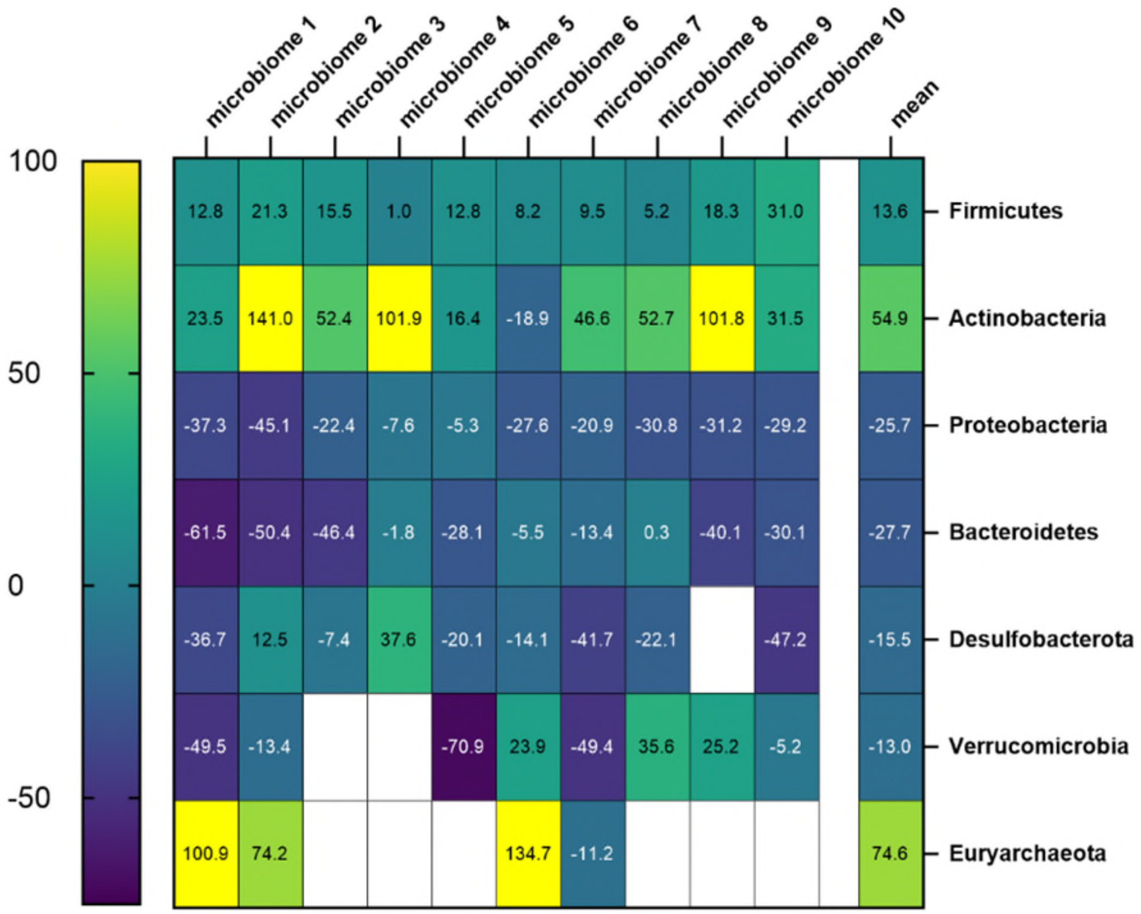

Supplementary Figure S1: Heatmap of fractional change in relative abundance induced by Menthacarin within the seven most abundant phyla (expressed in percentage) across 10 microbiomes. The numbers in each cell denote the fractional change (in %) with mean for each genus across 10 microbiomes shown in the final column.

Genus	microbiome 1	microbiome 2	microbiome 3	microbiome 4	microbiome 5	microbiome 6	microbiome 7	microbiome 8	microbiome 9	microbiome 10	Means
[Clostridium] innocuum group		18.7		28.3	217.8	71	27.8			-20.4	57.2
[Eubacterium] brachy group	-4.6	50.3		7.3					-25.5	88.7	23.2
[Eubacterium] eligens group			-70	73.1	9.4	-19.1	209.6	-42.9	25	79.2	33.1
[Eubacterium] hallii group	24	104.2	45.6	26.7	56.3	15.7	-6.1	24.9	53.8	75.4	42.1
[Eubacterium] nodatum group	-25.5	12.6	56.7	106.5	17.6	18.2	27.2	-14.8	-68	44.7	17.5
[Eubacterium] oxidoreducens group	-20.5	-14.7	-34.2	-44.3	19.9	11.6	-23.3	-8.6	-30.3	28.5	-11.6
[Eubacterium] ruminantium group		-48.5	-23.7			-73.9					-48.7
[Eubacterium] siraeum group			-61.5						23.3		-19.1
[Eubacterium] ventriosum group	-37	66.4				-6.2		292.8	65.1	-33	58
[Eubacterium] xylanophilum group								-38.3			-38.3
[Ruminococcus] gauvreauii group	-62.2	-80.1	-50.4	4.3	186.5	37.7	-35	4.5	93.4	49.5	14.8
[Ruminococcus] gnavus group	24.1	-50.7	111.3	37.5	11	6.4	-9.5	10.5	1.3		15.8
[Ruminococcus] torques group	29.9	-25.3	83.3	63.5	43.6	8.4	0.1	-7	162	5.9	36.5
28-4					-9.2						-9.2
Acetitomaculum									42.1		42.1
Acidaminococcus									-25.4	-47.7	-36.6
Actinobacillus		-5.9									-5.9
Actinomyces	68.5	260.1									164.3
Adlercreutzia		49.3			11.5	-41.4	30.6	8.6	-31.4		4.5
Agathobacter	-17.9	-19.7	25	7.3	39.3	9	49.2	30.5	39.6	-3	15.9
Akkermansia	-49.4	-13.4		-68.8	-71	29.8	-51.2	35.8	26.8	-5.1	-18.5
Alistipes	-65.6	-41.7	4.4	-20.7	-3.9	27.8	7.9	22.9	-40.2	-55.9	-16.5
Allisonella			29.6					-27.7			0.9
Alloprevotella			-76.1	-54.4							-65.3
Anaerofilum		90.4									90.4
Anaerostipes	8.7	9.4	-1.1	-4.6	47.3	15.3	5.7	22.6	45.6	-30.2	11.9
Anaerotruncus	-40.6	-6.6		113.6	141.4	31.2	118.5		-28.2	-14.8	39.3
Anaerovibrio			-67.3								-67.3
Asaccharobacter						-44			-34.5		-39.3
Bacteroidales bacterium 55.9						-89.7		-65.2			-77.4

Supplementary Figure S2 (1 of 6): Heatmap of fractional change in relative abundance induced by Menthacarin (expressed in percentage) across all identified 173 genera. The numbers in each cell denote the fractional change with mean for each genus across 10 microbiomes shown in the final column.

	microbiome 1	microbiome 2	microbiome 3	microbiome 4	microbiome 5	microbiome 6	microbiome 7	microbiome 8	microbiome 9	microbiome 10	Means
Bacteroides	-36.5	-44.3	17.2	-7.9	-7.9	-5.3	-13.5	5.7	-35.1	-27.6	-15.5
Barnesiella	-64.5	-72.1			76.5	1.3	-22.6	2.5	-31.9	39.2	-9
Bifidobacterium	31	159.2	234	117.4		-1.4	47.6	92.9	159.9	313.9	128.3
Bilophila	211.8	22.6	37.2	35.5	-20.7	-13.9	-41.6	26.8	-67.4	-49.9	14
Blautia	19.1	46.2	17.4	21.2	45.8	19.8	-15.7	-0.1	23	-33.8	14.3
Butyricicoccus	-51.5	12.8	-48.5	-0.4	-18.7	-43.2	21.4	-22	-70.9	338.2	11.7
Butyricimonas	-41.4	21.5		-20.8	-29	-31.6	-23.9	-3.7	-53.1	22.1	-17.8
CAG-352								-47.4			-47.4
CAG-56			-39.4	67.3	10.9	-41.5	16.2	-12.1	-20.5		-2.7
Candidatus Soleaferrea	-13.8	-6.8	69.4	-5	85	30.2	5	1.4	-41.5	70.7	19.5
Catenibacterium	43.6	201.4	10.4	-17.2	292.8	63.2	15.2	27.1			79.6
Catenisphaera		150.8	22.8		68.3	-13.4					57.1
CHKCI002									-65.1		-65.1
Christensenella		142.4									142.4
Christensenellaceae R-7 group	-11.5	-19.5	-4.9	28.5	45.7	-1.3	-10.7	18.8	20.2	146.9	21.2
Citrobacter						-20.3		102.7			41.2
Clostridiales bacterium 42.27						41.9		3.9			22.9
Clostridium sensu stricto 1	-0.2			-83.7				-65.4			-49.8
Colidextribacter	-20.8	-7.2	-60.2	-17	22.7	-11.5	1.7	-14.8	-5	113.9	0.2
Collinsella	16.1	45.4	238.2			-85.1				-15	39.9
Coprobacter									-77.2		-77.2
Coprococcus	19	-14.9	111.6	14	65.4	31.5	1.1	34.2	-13.9		27.6
Cupriavidus		-88	-55.8	-50.2	24.3	11.1	-64.8	10.6	156.6		-7
Defluviitaleaceae UCG-011								-60.3			-60.3
Desulfovibrio	-38.5	-69.5	-28.8			-0.3	-49.1	-83.9			-45
Dialister	-20		-28.3	-29.7		-2.5		15.9	32.5		-5.4
Dielma		-34.3								-54.3	-44.3
Dorea	19.9	-2	86.2	40.1	27.5	4.9	18.9	18.7	3.3	-0.1	21.7
DTU089		-51								-6.8	-28.9
Eggerthella		293.1		-28.1	-19	20.6	43.3	59.3	-50.3		45.6

Supplementary Figure S2 (2 of 6): Heatmap of fractional change in relative abundance induced by Menthacarin (expressed in percentage) across all identified 173 genera. The numbers in each cell denote the fractional change with mean for each genus across 10 microbiomes shown in the final column.

	microbiome 1	microbiome 2	microbiome 3	microbiome 4	microbiome 5	microbiome 6	microbiome 7	microbiome 8	microbiome 9	microbiome 10	Means
Eisenbergiella	83.5	-28.5		-57.1	-39.4	-3.9	-27.7	60.7	-47.5	214.7	17.2
Enterobacter		-9.4		-3.2	-32.9	-15.4		-68.3			-25.8
Enterorhabdus	-60.4		154.8	56.7		-49.1		4.3	-51.2	-20.3	5
Erysipelatoclostridium	87.3	172.1			68.3	-22.6		-5.6	364.9	150.5	116.4
Erysipelotrichaceae UCG-002	-11.4		-29.1								-20.2
Erysipelotrichaceae UCG-003	53.4	56.3		0.2	70.7	-8.6	13	80	81.7		43.3
Erysipelotrichaceae UCG-006			-0.3								-0.3
Escherichia	4.5	54.7	64.6	6.1	0.8	-7.1	20.5	-47.3	-23.2	17.3	9.1
Eubacteriaceae bacterium CH									-28.6		-28.6
Faecalibacterium	5	54.5	23.1	6.1	-2.7	-19.1	25.5	-21.6	-21.6	18.8	6.8
Faecalicoccus		120.1				40.1					80.1
Faecalitalea		157.8	29	47.9	81.9	-2					62.9
Family XIII AD3011 group	-22.2	-15.6	6.9	112.2	7.9	28.5	76.6	21.4	51.1	0.6	26.7
Family XIII UCG-001	-54.8	-54.2	86.4	3.4	30.3	2.8	-64.9	13.2	22.1		-1.7
Fenollaria	-83			29.6							-26.7
Flavonifractor		80.3	-12.5	-25.3	-41.9	-44.8	-58.9	-6.9	-44.1	-12.6	-18.5
Fournierella		-3.2	-44.5		-28.8	17		-7.4			-13.4
Frisingicoccus					79.4	25.5				-26.7	26.1
Fusicatenibacter	19.5	43.3	9.2	-50.3	-0.5	1.7	7.1	27.4	-24.9	36	6.9
Fusobacterium			-51								-51
Garicola									188.4		188.4
GCA-900066575	-38.5		-36.1		-85.2	48.8		24.1	13.5	9.1	-9.2
GCA-900066755		-85.9									-85.9
Gordonibacter		-16.7			55.7	3.1	-27	-8.4	-47.7	116.9	10.9
Hafnia-Obesumbacterium						-22.6		-8.3			-15.5
Holdemanella	13.3	31	29		40.3	28.6	3	1.5		52.2	24.9
Holdemania	65	-35.6		-8.6	51.5	-33	73	19.3	192.4	65	43.2
Howardella			-14.2		-33.4	60.4	5.3				4.5
Huakuichenia	35.4										35.4
Hungatella	-80.1			-17.9			-61.4			-7.5	-41.7

Supplementary Figure S2 (3 of 6): Heatmap of fractional change in relative abundance induced by Menthacarin (expressed in percentage) across all identified 173 genera. The numbers in each cell denote the fractional change with mean for each genus across 10 microbiomes shown in the final column.

	microbiome 1	microbiome 2	microbiome 3	microbiome 4	microbiome 5	microbiome 6	microbiome 7	microbiome 8	microbiome 9	microbiome 10	Means
Incertae Sedis	15.4	5.9	60.5	5.3	21.1	1.5	12.4	38.5	0	-34.2	12.6
Intestinibacter			-73.9	3.4	-4.4	88.9	28.7	14.3	1099.2	75	153.9
Intestinimonas	111.5	-42.3	-37.1	109.2	104.6	30.8	-7.5	185.1	72.7	205.3	73.2
Kandleria	77.1		-71.5								2.8
Lachnoclostridium	47.7	8.6	3	18.9	-23.1	0.2	-11.7	7.8	12.1	33.5	9.7
Lachnospira			-64.2	49.6	12.9	-13.2	533.7	-20.5	-20.7	57.7	66.9
Lachnospiraceae AC2044 group							-23.9				-23.9
Lachnospiraceae FCS020 group	-4.3		33.8	-28.1	31.5	-24.9	5.1	-27.4	-16	-4.6	-3.9
Lachnospiraceae NC2004 group	-32.6	-18.7	-11.8	-19.1	-27.5	-13.7	-9.9	4.3	27.4	30	-7.2
Lachnospiraceae ND3007 group		2.6	8.9	22.5	79.9	-8.3	165.4	-18.4	43.8	-61.2	26.1
Lachnospiraceae NK3A20 group			-6.5								-6.5
Lachnospiraceae NK4A136 group	86.5	47.4	16.8	-35.6	-2.4	-16.8	14.9	20.7	-6	76.3	20.2
Lachnospiraceae NK4B4 group								-72.8			-72.8
Lachnospiraceae UCG-001	38.8	35.4	-5.8		13	43.4	33.1	13.8	8.8		22.6
Lachnospiraceae UCG-004	-31.5	-55.9	5.1	-3.5	15.5	-9.9	57	54.3	37	-49.9	1.8
Lachnospiraceae UCG-006	-33.5	-48.8	16.3	-7.1	2.6	10.4	37.7	100.6	35.9		12.7
Lachnospiraceae UCG-008	27.8	46.2	14.7	9.8	-4.4	2.2	-7.4	6.4	19.3	11.9	12.7
Lachnospiraceae UCG-010	-6.8	16.1	-19.1	-13.6	22.8	46.6	12.7	10.3	-21.9		5.2
Lacticaseibacillus								28.5			28.5
Lactobacillus			139.4						-16.1		61.6
Lactococcus		50.8				17.9		15.1			27.9
Libanicoccus	6.8		132.5								69.6
Ligilactobacillus						13.5		55			34.3
Mailhella			-33.1								-33.1
Marvinbryantia	-15.3	-1.4	24.6	-10.7	-3.9	-10.6	26.7	-17.4	0.3	-18.1	-2.6
Megasphaera	-53.2		-100								-76.6
Merdibacter									-50.6		-50.6
Methanobrevibacter	100.9	70.6				151.9	-11.4	-31.4			56.1
Methanosphaera		92.4				84.3					88.4
Methylophaga				-65.5							-65.5

Supplementary Figure S2 (4 of 6): Heatmap of fractional change in relative abundance induced by Menthacarin (expressed in percentage) across all identified 173 genera. The numbers in each cell denote the fractional change with mean for each genus across 10 microbiomes shown in the final column.

microbiome 1	microbiome 2	microbiome 3	microbiome 4	microbiome 5	microbiome 6	microbiome 7	microbiome 8	microbiome 9	microbiome 10	Means	
		-47.3								-47.3	Mitsuokella
		-5.7	7.2	12.1	14.6					7.1	Mogibacterium
-56.1	214		24	140.7	13.6	9.8	22.5	-26.7	35.9	42	Monoglobus
-24.5	54.7	51.1	-40.2	80.3	47.1	-10.9	30.8	41.5	-40	19	Moryella
-23.2	-74.8	-21.2	-32.2	-18.5	19.4		-28.9	60.6	124.2	0.6	Negativibacillus
-42.3	18.9	-27.6	8.8	17	-13	22.7	40.4	25.8		5.6	NK4A214 group
-100	-94.8			-4.8	-30.9	-46.7	-11.4	-92.2	-66.4	-55.9	Odoribacter
2.4		20.6	-91.9							-23	Olsenella
		17	14.1		-31.6		83	-47.1		7.1	Oribacterium
54.1	45.3	7.6	10.7	7.7	1.8	-13.6	-13.3	47	36.5	18.4	Oscillibacter
	-47.8	15.3	-33.9	-55.5	-71.1	-14.6	-57.5	-29	-37.7	-36.9	Oscillospira
				-89.3		-87.2				-88.3	Paeniclostridium
-29.8										-29.8	Paludicola
-81.2	-59.1	104.2	13.9	-62.7	-11.1	-13.6	-19.1	-76.2	-19.8	-22.5	Parabacteroides
						-88.1				-88.1	Paraclostridium
				-89.2	-56.4	92.7	-84.5			-34.3	Paraprevotella
	-82.6	46.6	-41.3	-8.2	-21.2	-26.1	-25.2	135.6	-32	-6	Parasutterella
56.4			76.6					420.1		184.4	Peptoniphilus
			-7		-13.7		220.2	-28.7		42.7	Peptostreptococcus
	107.8	-30.6		18.3	-21.7	-12.4	-16.6	78.1	22.3	18.2	Phascolarctobacterium
-8.4	59	40.2	69.7	18.1	34.8	-10.9	13.7	91.9	-54.8	25.3	Phocea
-29.2	-73.9	-82.3	-70.9	-35.6	-75.1	47.3	-84.3	-72.2	100.9	-37.5	Prevotella
-84.9	-65.4	-59.4							-100	-77.4	Prevotellaceae NK3B31 group
		-62.1	-88.5							-75.3	Prevotellaceae UCG-003
-84.8			115.1							15.1	Raoultibacter
		-87								-87	Rikenellaceae RC9 gut group
122.9	16	-74.1	-16.4		-1.2	-45.4	-16.4			-2.1	Romboutsia
5.5	-31.9	42.1	-6.6	51	16.6	36.9	17.3	-0.7	59.6	19	Roseburia
-3	-14.9	25.6	-8.2	10.1	14.5	26.4	-0.6	39	32.9	12.2	Ruminococcus
-78.5			31.3						40.7	-2.2	S5-A14a

Supplementary Figure S2 (5 of 6): Heatmap of fractional change in relative abundance induced by Menthacarin (expressed in percentage) across all identified 173 genera. The numbers in each cell denote the fractional change with mean for each genus across 10 microbiomes shown in the final column.

	microbiome 1	microbiome 2	microbiome 3	microbiome 4	microbiome 5	microbiome 6	microbiome 7	microbiome 8	microbiome 9	microbiome 10	Means
Selenomonadales bacterium M					49.4	-16.6					16.4
Sellimonas		110.8			120.3	27.6			-88.5	-75.2	19
Senegalimassilia	19.3		-4.9	-24.1		-35.4	32.7	-3.6		36.4	2.9
Serratia				14.3							14.3
Shuttleworthia									-42.1		-42.1
Slackia	-69.7		15.4		33.3	-58.6		50.5	54.7	6.1	4.5
Solobacterium			69	148.3							108.6
Streptococcus	-31.6			54.9	6.1	10		1.2	53.6		15.7
Subdoligranulum	26.5	23	0.6	-31.8	-5.5	-22.4	19.3	-21.9	47.8	52.6	8.8
Succinivibrio			-33.1								-33.1
Sutterella	-60.3	-90.5	-52.3	-19.1	-13.2	-32.1	-26.5	-31.4	-45	-79.1	-44.9
Terrisporobacter								-67.6			-67.6
Turicibacter		-4.5									-4.5
Tuzzerella				-35.8						-13.1	-24.5
Tyzzerella		0.1		-18.3	-60.5	-6.9	0.7	6.4		-8.1	-12.4
UBA1819	-17.5										-17.5
UC5-1-2E3					-48	-48.7					-48.4
UCG-002	-30.9	24	79.8	-18.5	28.9	-1.5	8.9	0.8	-1.4	-3.8	8.6
UCG-003	-39	-68.9	-39.4	-9.4	-25.1	0.6	-28.8	-15.4	-4.1	-33.4	-26.3
UCG-005	-18.5	37.3	-14.4	-17.9	2.4	-11.1	0.8	24.7	-27.4		-2.7
UCG-008									-54.3		-54.3
UCG-009	-70.7	26.6	10.5	18.9	29	4.1	-54.6	-33.2	32.7		-4.1
Victivallis	-59.3							86.1			13.4

Supplementary Figure S2 (6 of 6): Heatmap of fractional change in relative abundance induced by Menthacarin (expressed in percentage) across all identified 173 genera. The numbers in each cell denote the fractional change with mean for each genus across 10 microbiomes shown in the final column.

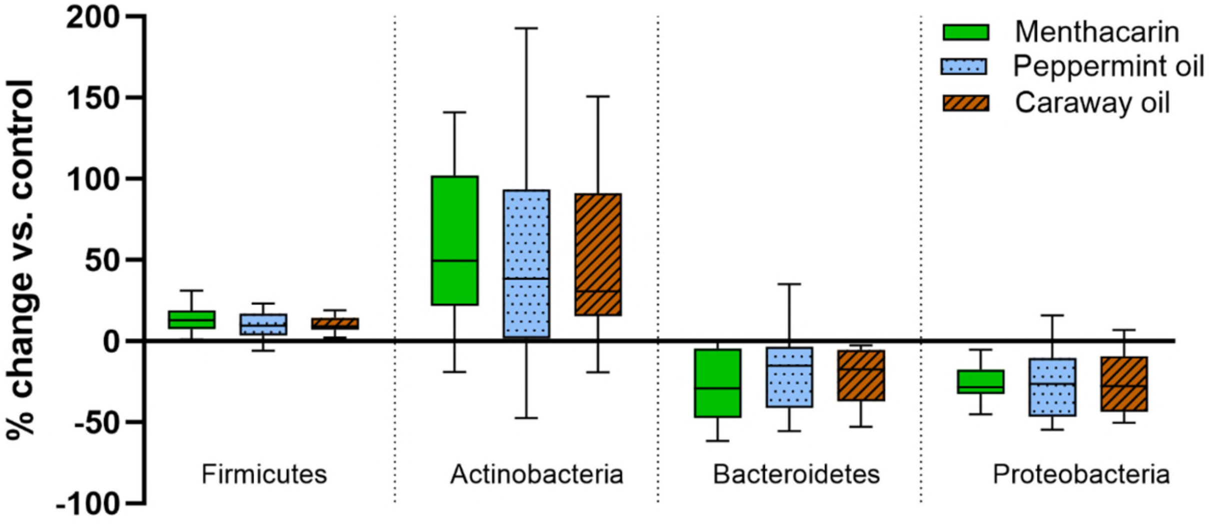

Supplementary Figure S3: Peppermint and caraway oil induce similar fractional changes in relative abundance of the phyla that are significantly affected by Menthacarin. Data are presented as box-and-whiskers plots with quartiles and min-max values.

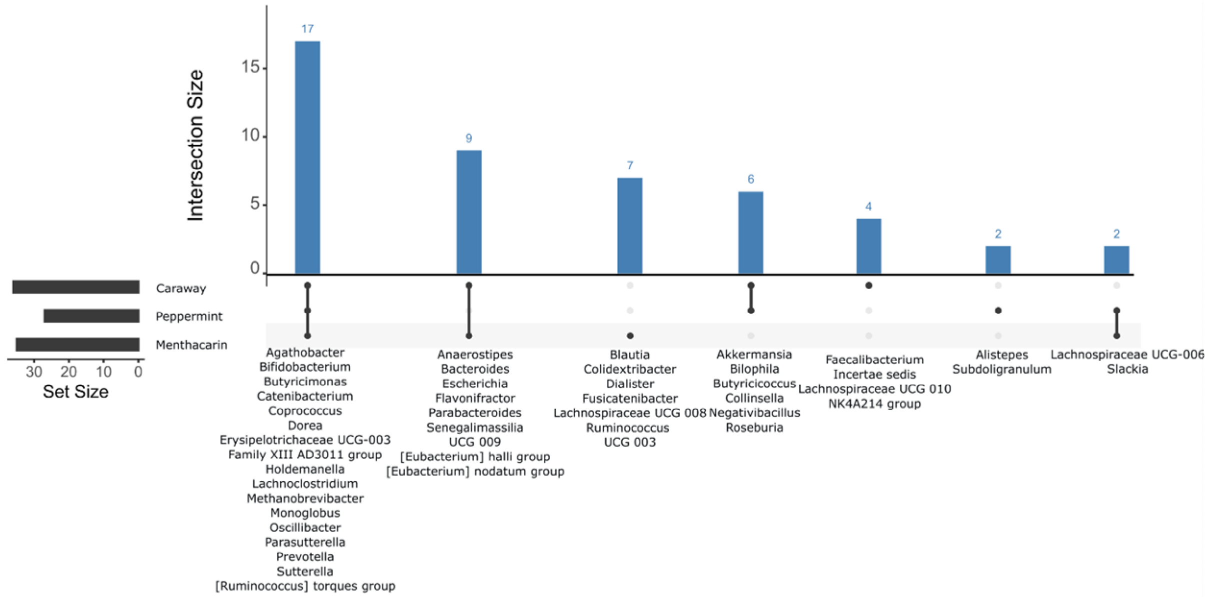

Supplementary Figure S4: UpSet plot showing the proportions of genera exhibiting consistent fractional change with treatment of Menthacarin, peppermint oil, and caraway oil in at least 70% of tested microbiomes. Genera present in at least 4 microbiomes were considered.
